# Advancing aesthetic outcomes: a critical analysis of autologous free flap reconstruction post-minimally invasive mastectomy

**DOI:** 10.1097/JS9.0000000000002087

**Published:** 2024-10-08

**Authors:** Qirui Guo, Mohan Liu, Yan Li, Qiang Sun

**Affiliations:** a Department of Critical Care Medicine, Peking Union Medical College Hospital, Peking Union Medical College & Chinese Academy of Medical Sciences; b Department of Breast Surgery, Peking Union Medical College Hospital, Peking Union Medical College & Chinese Academy of Medical Sciences, Beijing, China

*Dear Editor,*

We are highly interested in Wong *et al*.’s^1^ research, which explores the feasibility of aesthetic reconstruction using autologous free flaps after minimally invasive mastectomy. This study demonstrates a profound understanding of surgical details through six key steps and reflects modern plastic surgery’s innovation in integrating aesthetics with functional recovery. It provides valuable clinical insights and technical guidance crucial for improving patients’ quality of life and self-confidence. Clinically, we aim to conduct an in-depth analysis of this technique to enhance its recognition in practice.

Minimally invasive mastectomy suits early breast cancer, high-risk patients, and those opting for prophylactic mastectomy. Contraindications include larger tumors, cancer spread, and poor health. Using small incisions and endoscopic technology, this surgery reduces skin and tissue damage, minimizing postoperative pain and discomfort. Often, the nipple–areola complex is preserved, benefiting patients’ psychological health and body image. Smaller incisions allow personalized procedures but complicate microvascular anastomosis and flap shaping. Anastomosis of thoracodorsal or lateral thoracic vessels may require retractors or assistants due to limited exposure. Accurate flap volume and vascular length design are crucial to avoid poor contour or anastomosis difficulties. Oversized flaps may not fit well, and undersized ones may not fill the space adequately. Shaping and fixing flaps through narrow incisions demand repeated adjustments, increasing surgical time and complication risks, such as vascular tearing. Despite reduced visible scarring, aesthetic revisions like fat grafting or contralateral breast balancing may still be necessary due to high aesthetic expectations.

Although the study reported a high flap survival rate, it lacked a detailed analysis of surgical complications and failure rates. The paper emphasizes optimizing flap perforator selection and design through preoperative angiography and Doppler studies. However, preoperative imaging results may differ from intraoperative conditions, affecting surgical accuracy and outcomes. The resolution and accuracy of angiography and Doppler ultrasound are limited, especially in imaging small vessels and capillaries, potentially causing discrepancies between preoperative assessments and actual conditions. Additionally, patients’ vascular conditions may change during surgery, such as variations in blood pressure and volume, affecting vessel filling and visibility. The operator’s skill level and experience also impact accuracy. To address these issues, higher-resolution imaging technologies like 3D ultrasound, CT angiography (CTA), and magnetic resonance angiography (MRA) can provide more detailed vascular images^2^. Introducing intraoperative real-time imaging techniques, such as fluorescent angiography (ICG) and Doppler ultrasound, can monitor vessels and blood flow in real-time. Using computer-aided design (CAD) and 3D printing for preoperative simulation and planning, combining multiple imaging techniques for comprehensive vascular analysis, and enhancing operator training and standardized procedures can significantly improve the accuracy of preoperative evaluations, ensuring precise flap design and harvest, thereby optimizing surgical outcomes and improving patient satisfaction.

Aesthetically, although this study emphasizes the importance of outcomes, it mainly relies on subjective assessments, limiting result consistency. Quantitative evaluation and objective aesthetic scoring systems are crucial for successful breast reconstruction surgery. Previous studies have used objective scoring systems, like 3D imaging technology, to quantify breast volume, symmetry, shape, and position, providing standardized assessment methods^3^. Introducing these systems enhances comparability and helps determine the best surgical strategies. Similarly, patients’ satisfaction and quality of life are crucial for evaluating surgery effectiveness. Future research should use long-term follow-up and standardized questionnaires to assess psychological adjustment, sexual satisfaction, pain perception, and changes in self-image^4^. These indicators reflect the objective effects of surgery and its comprehensive impact on patients’ quality of life, providing important standards for selecting and optimizing surgical methods^5^.

Finally, although short-term to mid-term follow-up data provide a preliminary understanding of surgical outcomes, long-term follow-up data are essential for evaluating the durability of the results, stability of patient satisfaction, and the risk of long-term complications^5^. Therefore, long-term follow-up should be conducted to assess the aesthetic and functional outcomes of the breast and the possibility of secondary corrective surgeries. This is crucial for understanding the long-term value of surgical techniques and guiding future adjustments and improvements in surgical strategies. By adopting this approach, we can ensure continuous technological improvement to better serve patients undergoing post-mastectomy reconstruction surgery.

In conclusion, while this study significantly advances aesthetic reconstruction post-minimally invasive mastectomy, its potential is underutilized and needs improvement. Objective aesthetic scoring and detailed patient satisfaction assessments will yield more reliable evaluations. Analyzing surgical complications and technique adaptability across various settings is crucial. Emphasizing long-term follow-up will provide valuable insights into the surgery’s lasting effects. Addressing these issues will enhance research outcomes and advance post-mastectomy reconstruction, ultimately improving patient well-being.

## Ethical approval

As this letter to the editor does not involve research or data collection from patients, ethical approval is not applicable.

## Consent

As this letter to the editor does not involve patients or volunteers, informed consent is not applicable. There are no patient or volunteer data, images, or case reports included in this submission. Therefore, no explicit consent statement is required. Additionally, no identifying details of patients or volunteers are disclosed in this letter.

## Source of funding

This work was supported by a grant from the *Non-profit Central Research Institute Fund of Chinese Academy of Medical Sciences(#2023-RW320-07)*.

## Author contribution

Q.G.: carefully analyzed the details of the research, identified and proposed innovative points, and took full responsibility for drafting the letter; M.L.: thoroughly examine the details of the research, help discuss and determine the groundbreaking aspects, and take partial responsibility for drafting this letter; Y.L.: guide the surgical thoughts and provide a comprehensive review of this article; Q.S.: support as Breast Surgery Aesthetic Consultant.

## Conflicts of interest disclosure

The authors declare no conflicts of interest.

## Research registration unique identifying number (UIN)

This article does not involve research registration, as it is a letter to the editor and does not include any research involving human participants. Therefore, no unique identifying number (UIN) from a registration body is applicable to this submission.

## Guarantor

The guarantor for this letter is Professor Yan Li, who takes full responsibility for the conduct of the work, has access to the data, and controls the decision to publish.

## Data availability statement

This letter to the editor does not involve the generation or analysis of research data. Therefore, data availability is not applicable to this article.

## Provenance and peer review

This manuscript has not been commissioned for external peer review.
